# Characterizing Cochlear implant artefact removal from EEG recordings using a real human model

**DOI:** 10.1016/j.mex.2021.101369

**Published:** 2021-04-25

**Authors:** Jaime A. Undurraga, Lindsey Van Yper, Manohar Bance, David McAlpine, Deborah Vickers

**Affiliations:** a Department of Linguistics, 16 University Avenue, Macquarie University, NSW 2109, Australia; b Department of Clinical Neurosciences, Cambridge Biomedical Campus, University of Cambridge, CB2 0QQ, UK

**Keywords:** Electrical artefact, Cochlear implant, DSS, Spatial filter, Interpolation, Inter-DSS, ASSR, ACC, AC-FR, Steady-state, eASSR, eACC, eAC-FR

## Abstract

Electroencephography (EEG) recordings from CI listeners are contaminated by electrical artefacts that make it difficult to extract neural responses. Previously, we have removed these artefacts by means of interpolation and spatial filtering. However, the extent to which this method can effectively reduce electrical artefacts has not been fully investigated. Here, we assessed the effectiveness of interpolation and spatial filtering to remove electrical artefacts using recordings from a human head specimen implanted with a CI.

• Electrical artefacts were obtained using amplitude-modulated (AM'ed) pulse trains presented at several pulse rates (100-to-902 pps) or using high rate pulse trains (902 pps) in which either a pair of electrodes or AM frequencies alternated periodically at a rate of 1Hz.
• By adding auditory change complex (ACC), auditory steady-state response (ASSR), or auditory change following response (AC-FR) template waveforms to the contaminated recordings, we show that interpolation allows for effective artefact removal for pulse rates below 400 pps whilst interpolation and spatial filtering are effective at higher pulse rates, with minimal distortions for ACC and AC-FR, and with a degree of amplitude- and phase-distortions for ASSR.
• Recordings from CI listeners agreed with simulations, demonstrating that reliable responses can be recovered.

## Graphical abstract

**Figure:**
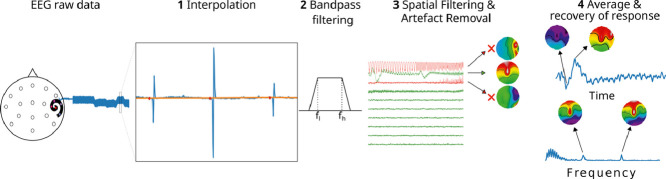

Specifications table

**Table:** 

Subject Area:	Neuroscience
More specific subject area:	Auditory evoked neural responses
Method name:	Interpolation and spatial filtering artefact removal
Name and reference of original method:	[1,[3], [4], [5]]
Resource availability:	Software: https://gitlab.com/jundurraga/pyeeg-python/ Data and analysis scripts available on request

## Methods

Electroencephography (EEG) recordings to electrically evoked sounds by cochlear implants CIs are challenging due to the presence of large electrical artefacts caused by electrical stimulation. This is particularly difficult when stimuli are presented at clinical stimulation rates—≈ 900 pulses per second (pps)—in which both temporal and spatial (different EEG electrodes) recordings are strongly contaminated.

The next set of experiments aimed to investigate the extent to which electrical artefacts could be removed from realistic recordings under electrical stimulation. To this end, a cadaveric human head was implanted with a CI and EEG recordings were made under several conditions, described in the next sections. These recordings allowed us to characterize several types of realistic electrical artefacts in a controlled environment. Furthermore, a large number of simulated neural responses were imposed to these recordings allowing us to investigate the advantages and limitations of the artefact removal method used in this study. The method combines interpolation—blanking of electrical artefacts via linear interpolation between time samples preceding the electrical pulses [1]— and denoising source separation (DSS) —a spatial filter that partitions components into highly and weakly reproducible ones [2].

The large number of conditions tested in this study would have been impossible to carry out in human CI listeners. However, examples from CI listeners are also provided in this study and fully reported in the associated research article [3].

Cadaveric recordings were conducted at the University of Cambridge. This was approved by the ethical board of Cambridge University (19/NE/0366) and complied with the Human Tissue Act (2004).

### Implantation

The cadaveric head of a 74-years-old male human was implanted in the right cochlea with a Cochlear electrode array (CI522). The sample was fresh frozen, without any preservatives to preserve the tissue in optimal conditions, and it was defrosted within 24 h of the experiment. The CI was fully inserted (round window approach) and the cochlea was flushed prior to implantation using a 1% saline solution in order to remove air bubbles.

The quality of the electrodes was assessed by impedance measures which were ≈ 2kΩ for the electrodes tested (15 and 16).

EEG recordings were obtained using a 32-channel high resolution BioSemi ActiveTwo system (Amsterdam, The Netherlands) at a sampling rate of 16384Hz and a resolution of 24 bits/sample (31 nV LSB). Channels were arranged according to the international 10–20 system. Voltage offsets were within ±20 mV and EEG data were referenced to the channel contralateral to the implant (T7). This reference electrode was selected to allow direct comparisons with the associated research article [3] where a contralateral electrode, relative to the CI, was used as the reference. In that study, as well as previous ones [4], [5], we have found that contralateral electrodes show significantly less artefacts than electrodes in close proximity to the CI. Therefore, using a contralateral reference minimized the spread of electrical artefacts offering an advantage for real-time assessment, since it is often sufficient to unveil artefact-free responses on contralateral frontal electrodes. Note that the use of a contralateral electrode as the reference has no impact on the quality of the responses investigated in this or previous studies [3], [4], [5].

Due to the use of the cadaveric specimen, for which the CI needed to be implanted using specialized surgical equipment, all recordings were obtained in a standard clinical booth (i.e. not electromagnetically insulated).

### Stimuli

If not stated explicitly, all stimuli consisted of quadra-phasic-cathodic (QP-C) pulses with 43μs phase width and 7μs interphase gap presented in monopolar mode (MP 1+2). Monopolar stimulation was preferred as it is most commonly used in the clinic. Furthermore, monopolar stimulation offers a worst case scenario in terms electrical artefact contamination in the spatial- (across electrodes contamination) and temporal-domain (post-stimulus contamination limiting artefact interpolation; [1]). Different pulse rates were used spanning between 100 and 902 pps. Low pulse rates were chosen to investigate the effectiveness of interpolation on its own, i.e. without DSS, whilst high pulse rates were used to investigate the ability to remove electrical artefacts at clinical pulse rates. The stimulation level was 180 CU in all recordings. A total of 241 epochs of ≈ 1s were presented per condition.

### Cochlear implant artefact suppression

**Fig. 1:**
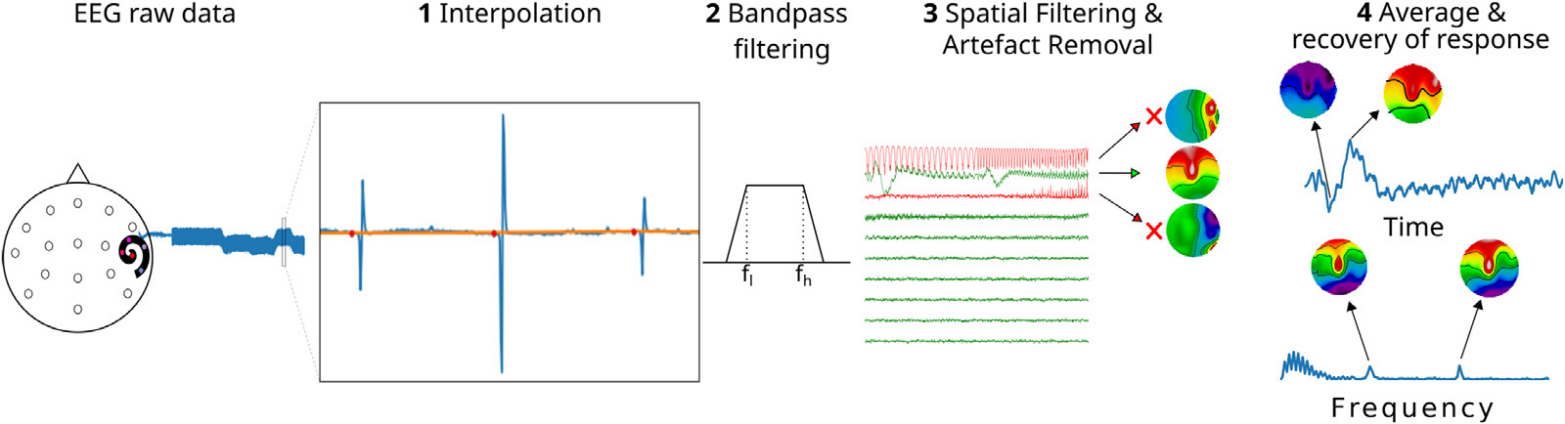
General framework for artefact reduction including **1** linear interpolation of the raw data (original data in blue; interpolated data in orange; interpolating points in red), **2** bandpass filtering, **3** spatial filtering and artefact suppression across epochs via DSS. The removed components are indicated in red and their respective topographic maps are indicated by the arrows. **4** Recovery of neural responses via averaging of de-noised epochs in the time- and frequency-domain with their respective topographic maps.

In previous studies, we have successfully removed electrical artefacts from auditory change complex (ACC) responses—a cortical response evoked by transitions of auditory cues and characterized be a positive peak (P1) around 50 ms, a negative peak (N1) around 100 ms, and a positive peak (P2) around 200 ms [4], [5]. This success has been, in part, based on the fact that linear interpolation (illustrated in Fig. 1) reduces the strength of those artefacts as well as presence of non-linear components in the recordings significantly. This in turn facilitates the identification and removal of residual artefacts via DSS. DSS partitions the data into stimulus-related and stimulus-unrelated components (for details see [2]). This offers an advantage to identify artefact and neural components, both of which are strongly reproducible but emitted from spatially differentiable sources, whilst, at the same time, facilitating the elimination of stimulus-unrelated components such as heart activity, eye-blinks, and ongoing brain activity.

The general steps of the artefact removal framework used in this study (Fig. 1) included linear interpolation of electrical artefacts, bandpass filtering (2–47 Hz; non-causal zero-phase third-order finite impulse response (FIR) Kaiser filter), spatial filtering and artefact elimination via DSS, and the recovery of the neural response via weighted average [6]. All signal processing was carried out off-line using a custom analysis module “pyeeg-python” developed in Python 3.

### Neural response simulation

To fully characterize the ability to remove electrical artefacts we simulated and added “neural responses” to the recorded artefacts. Three type of neural responses were simulated: transit ACC responses—a cortical response evoked by transitions of auditory cues at a low rate (e.g. 0.5 Hz)—; ASSRs—a neural response that follows the periodic amplitude modulations (AMs) of a stimulus (e.g. 40 Hz; [7])—; and steady-state auditory change following response (AC-FR)—a cortical response evoked by transitions of auditory cues at a high rates (e.g. 7 Hz; [3], [8]). ACC responses were generated using a template response with P1-N1-P2 peaks. Their amplitudes were set so that the maximum peak-to-peak N1-P2 amplitude was 4 uV across the scalp, a value that corresponds well with those obtained in real measurements (e.g. [4]). ASSRs consisted of a sinusoidal signal with a carrier frequency that was identical to those delivered by the CI in the cadaveric recordings. The AM frequency was jittered by 1% and the maximum ASSR amplitude was set to 500 nV across the scalp. For all simulations, the artefact-free neural response was added to the already referenced cadaveric data (T7 electrode) and it was scaled across the scalp so that large neural activity was found in frontal electrodes and small activity towards the back of the head, similar to the topographic distribution observed in CI users when the reference electrode was the contralateral mastoid (Fig.3C & Fig.4A; [4], [5]). Note that adding the simulated neural data to the already referenced cadaveric data had no effect for any of the analyses presented in this study, other than allowing us to produce a desire scalp distribution more easily.

As the focus of this work was to characterize the ability to separate electrical artefacts from stimulus-related neural responses, we do not report simulations of stimulus-unrelated noise, and it was assumed that poor electrodes, eye-blinks, or other endogenous sources have been already suppressed. It was also assumed that the average neural response had a positive signal-to-noise ratio (SNR). Unreported simulations including eye-blinks and non-stationary noise (i.e. negative SNRs) confirmed that the results of this study remain valid providing that the processing pipeline includes weighted average and DSS. Note, however, that cadaveric recordings were largely contaminated by both stimulus-related and stimulus-unrelated electrical noise due to the recording environment.

### Electrical artefacts at different stimulating electrode locations

**Fig. 2:**
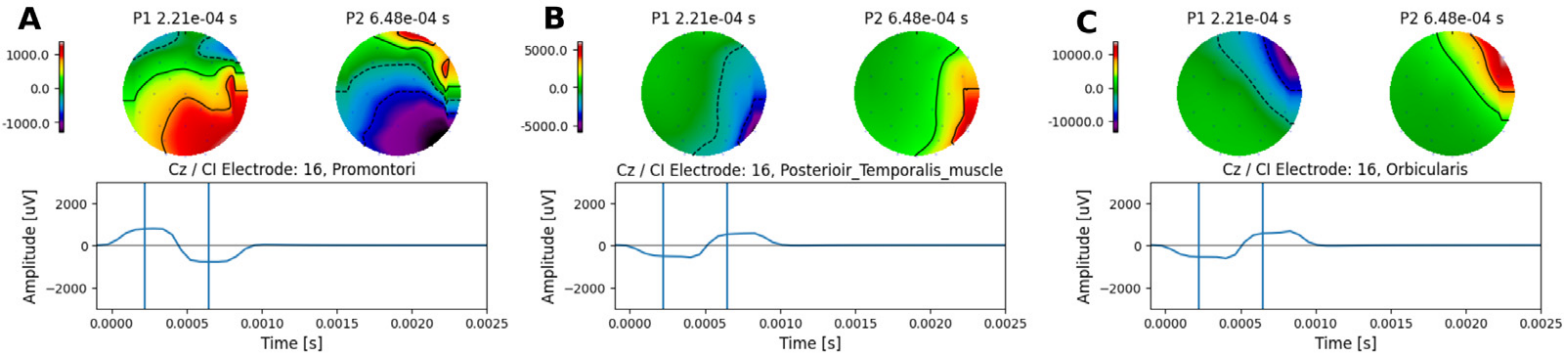
Topographic maps at different reference electrode (MP1) locations. **A** On the promontori, **B** posterior medial of the temporal muscle, and **C** near orbicularis oculi muscle. Topographic maps were obtained for two time points, at the middle of each pulse phase, shown by vertical lines alongside the waveforms.

First, we investigated the extent to which topographic maps are indicative of artefact contamination. This was achieved by placing the CI return electrode (MP1) at three different locations and recording the electrical artefacts at each of these locations. Monopolar symmetric biphasic pulses with a long phase width (400μs per phase, and 7μs interphase gap) were presented at a low rate of 35 pps. The return electrode (MP1) was placed on three different locations: 1) the promontori, 2) the posterior medial of the temporal muscle, and 3) near orbicularis oculi muscle (lateral edge of orbicularis oculi right eye). As expected, the topgraphic maps changed with the location of the return electrode (Fig. 2), confirming that the use of the topographical distribution and the known location of the implanted CI provide relevant information about the presence of residual artefacts.

### Effectiveness of interpolation and spatial filtering in the removal of artefact in ASSRs

Here, we assess the extent to which artefact removal can be achieved via interpolation and DSS in ASSRs.

To this end, we recorded artefacts produced by 40 Hz 100% amplitude-modulated (AM’ed) pulse trains (between 0 and 180 CU) using pulse rates between 100 and 902 pps.

**Fig. 3:**
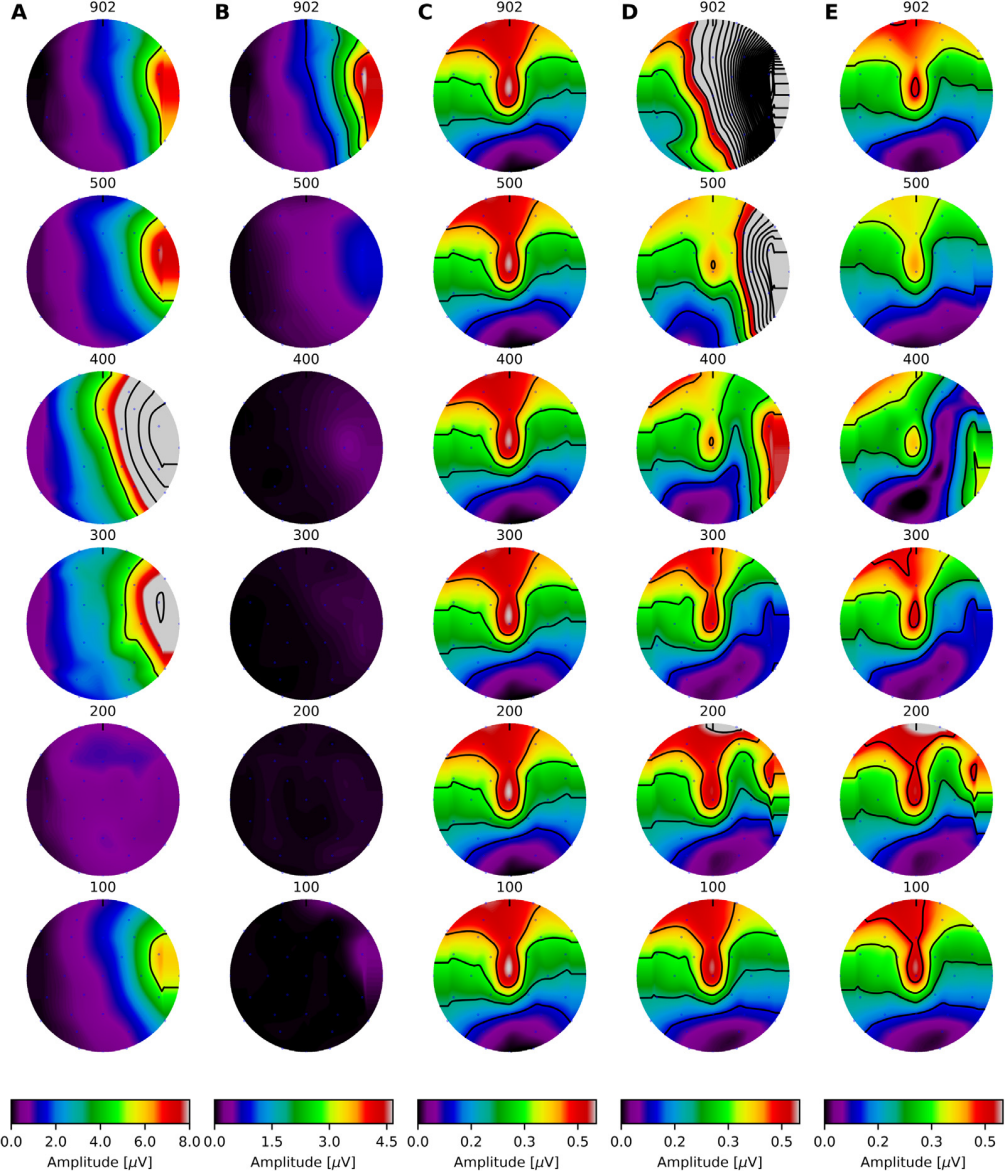
Topographic maps for ASSR amplitudes at different pulse rates and interpolation time points. **A** Electrical artefacts obtained by interpolating between the onset of each electrical pulse (strong artefact contamination), and **B** interpolating between points located one eight of the pulse rate period, before the pulse onset (minimum artefact contamination). **C** Topographic map of simulated ASSR; **D** topographic map of contaminated response (**B** + **C**); and **E**, topographic maps of recovered neural response. The amplitude of the 40Hz frequency component at each recording electrode is colour coded and indicated by the colorbar in each panel. Note that the maximum value shown in the colour scales was limited to facilitate visualisation across conditions, however, residual artefacts exceeded 24uV in many cases.

First, we proceed by interpolating the electrical artefacts starting at two different time points (the interpolation length was fixed and equal to the pulse rate period). In the first case, interpolation was applied between points located at the onset of each electrical pulse—likely contaminated by strong electrical artefacts—and, in the second case, interpolation was applied between points located before the onset of the pulse (one eight of the respective pulse rate period), where we expected an optimal artefact cancellation. In agreement with previous studies, interpolation was inefficient when the starting point was too close to the electrical pulse (Fig. 3A), but it was optimal when the starting point preceded the electrical pulse for pulse rates between 100 and 500 pps for electrodes located contralaterally to the CI, and in all electrodes for rates below 400 pps (Fig. 3B; Hofmann and Wouters [1], Luke et al. [9], Deprez et al. [10]).

**Fig. 4:**
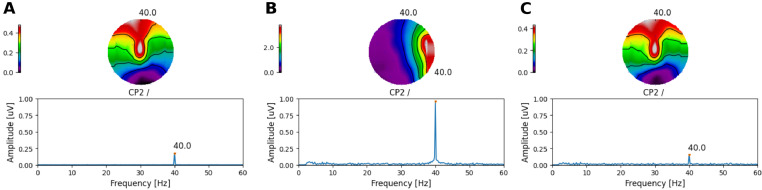
Topographic maps for ASSR at 902 pps. **A** Simulated neural response, **B** recorded electrical artefact plus simulated neural response, and **C** recovered neural response. Topographic maps were obtained for the ASSR frequency response (40 Hz). The response amplitude at each recording electrode is colour coded and indicated by the colorbar in each panel.

To fully characterize the recovery of ASSRs from contaminated recordings, we simulated and added “neural responses” to the recorded artefacts. This allowed us to quantify the effect of artefact removal on otherwise highly temporally correlated conditions (Fig. 4A & B). ASSRs frequencies were identical to those delivered by the CI and the phase was parametrically varied between 0${}^{\circ }$ and 360${}^{\circ }$ in steps of 22.5${}^{\circ }$.

The results showed that interpolation works well, i.e. with minimal distortions, for pulse rates below 400 pps across all recording electrodes (cf. Fig. 3C & D). As the pulse rate increased from and above 400 pps, artefacts started dominating the recordings obstructing the detection of the neural response. Removing residual artefact components via DSS allowed the recovery of the ASSR across all pulse rates (Fig. 3E & Fig. 4C). Only a minor residual component was observed at 400 pps on the right side and it was caused by the similarity between neural and artefact amplitudes, which made their separation difficult.

To further investigate the relative effect of the ASSR phase on the recovered response at 902 pps via interpolation and DSS, a total of 16 different starting phases, equally separated in steps of 22.5${}^{\circ },$ were simulated. Interpolation between consecutive pulses started at one eight of the pulse rate period, before the onset of each pulse.

**Fig. 5:**
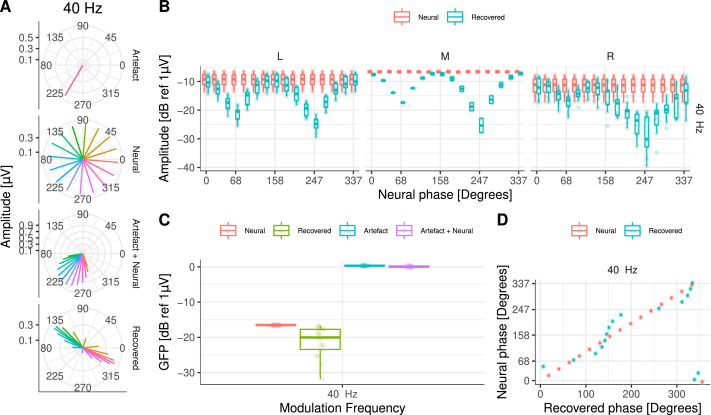
**A** Amplitude and phase of the 40 Hz ASSRs at Cz. Each panel shows the amplitude and phase of: artefact only, target (neural), artefact plus neural, and recovered ASSRs, respectively. The different phases of the neural source are colour coded. **B** ASSR amplitudes for central and frontal recording electrodes on the left (L), middle (M), and right (R) side of the head. **C** GFP for all simulated ASSRs. Target (neural), recovered, artefact only, and artefact plus neural data are colour coded. **D** Target (neural) and recovered phase (in degrees) for simulated ASSRs at Cz.

The effect of the electrical artefact (Fig. 5A, Artefact panel) on the target neural response (Fig. 5A, Neural panel) resulted in a distorted amplitude and phase in which the electrical artefact “drags” the neural response towards its direction (Fig. 5A, Artefact + Neural panel). By removing the DSS component with the largest energy on the side of the CI device, the amplitude of the recovered ASSR was reliably for many of the simulated neural phases across recording electrodes (Fig. 5B). However, the phase and amplitude of the recovered responses were distorted. For some specific neural phases, the recovered amplitudes were smaller and their phases were biased towards directions orthogonal to the artefact (Fig. 5A, Recovered panel, B, & D). The estimated global field power (GFP) to each simulated condition (Fig. 5C) indicated that the average difference between the neural (−16.5dB ± 0.03dB) and the recovered GFP (−21.3dB ± 4.40dB) was 4.8dB.

These results demonstrate that ASSRs can be recovered from highly contaminated recordings at low pulse rates via interpolation and at high pulse rates via interpolation and DSS artefact removal. However, the amplitude may result suppressed and the phase biased when DSS is applied, depending on the specific relationship between the neural phase and the artefact phase. It should be noted that the recovered topographic map was similar to that of the neural source across all conditions and, therefore, it can be used as an indication for the presence of a genuine neural response.

### Effectiveness of interpolation and spatial filtering in the removal of artefacts caused by AM frequency and electrode alternations

**Fig. 6:**
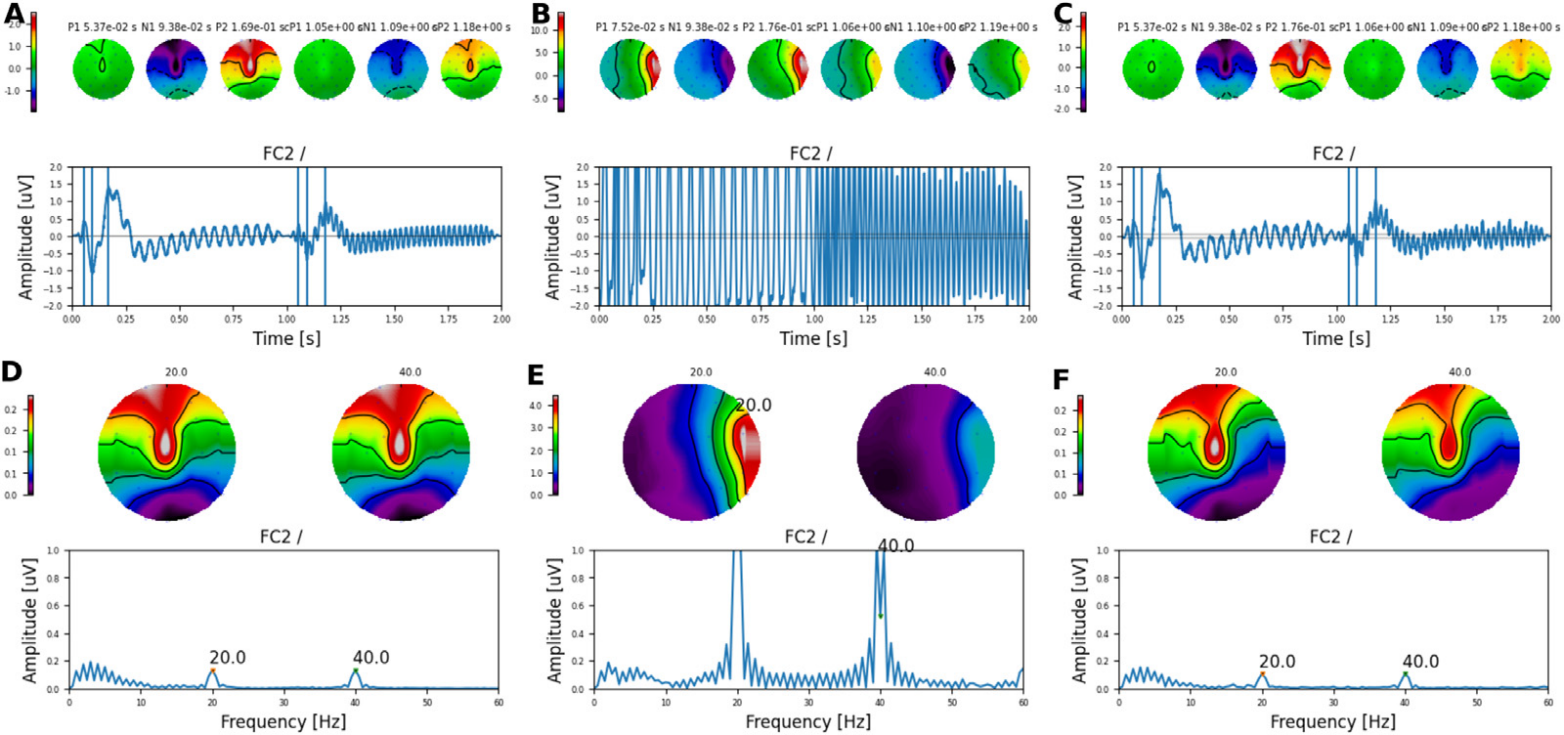
Topographic maps for AM frequency alternating simulations. **A** Target neural response, **B** artefact plus neural response, and **C** recovered neural response. Topographic maps were obtained for the time points shown by vertical lines alongside the waveforms. **D, E**, and **F** same as before but in the frequency-domain.

To fully characterize the limitations of our artefact rejection method, we recorded the electrical artefact from the cadaveric specimen using the AM frequency alternating paradigm. In this paradigm, two AM frequencies periodically alternate evoking either an ACC response when this alternation occurs at a low rate (e.g. 0.5 Hz) or an steady-state AC-FR when this alternation is delivered at a higher rate (e.g. 7 Hz). In addition to these cortical responses, ASSRs to each AM frequency are also evoked [3]. Here, the pulse train was presented at 902 pps and the AM frequencies (20 Hz and 40 Hz) were presented at an alternating rate of 1 Hz on the CI electrode 16. As in the previous section, we simulated and added “neural responses” both ACC and ASSRs to the recorded data. A template ACC response was imposed after each AM transition, each generating an N1-P2 complex (referred as to cN1-cP2 when generated by the second AM). The maximum N1-P2 amplitude was set to 4 uV across the scalp and the topographic distribution was identical to that used in the previous section. To assess the representation of relative neural contributions, the ACC amplitudes to each AM differed by half (Fig. 6A). We also simulated ASSRs to each AM frequency, both having the same amplitude (Fig. 6A & D).

**Fig. 7:**
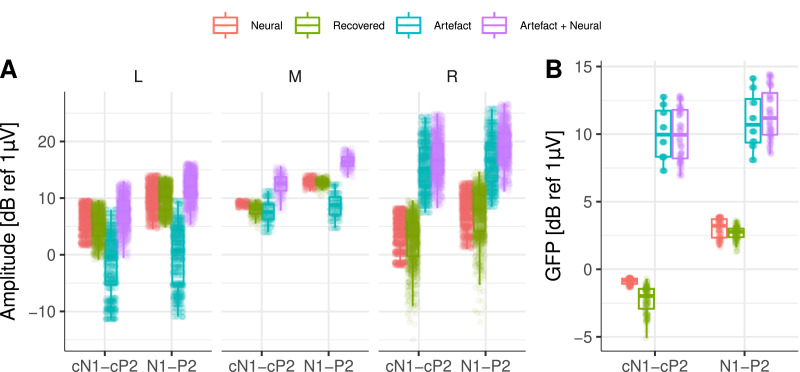
Amplitude and GFP across all simulated conditions. **A** ACC amplitudes for the first (N1-P2) and second (cN1-cP2) simulated responses for central and frontal recording electrodes on the left (L), middle (M), and right (R) side of the head. **B** GFP for the first (N1-P2) and second (cN1-cP2) simulated ACC responses. Target (neural), recovered, artefact only, and artefact plus neural data are colour coded.

As in the previous section, we investigated the relative effect of interpolation and ASSR phase on the recovered response, a total of 16 different neural phases were simulated equally separated in steps of 22.5${}^{\circ },$ and 8 different equally spaced starting interpolation points (between zero and half the pulse rate period, before the onset of each pulse), yielding a total of 128 different simulations. The reliability of the recovered ACC responses was excellent across all simulations (cf. Fig. 6A-F). The difference between neural and recovered amplitudes or GFP (Fig. 7A & B) was small. The GFP for the neural responses, i.e. N1-P2 and cN1-cP2, was 3.04dB ± 0.70dB and −0.89dB ± 0.20dB, respectively (3.94dB difference), whilst the recovered GFP at N1-P2 and cN1-cP2 was 2.68dB ± 0.46dB and −2.25dB ± 0.95dB, respectively (4.93 dB difference). This resulted in relative differences between neural and recovered response of ≈ 1dB and confirmed the reliability of our artefact removal method for ACC responses in CI listeners [3], [4], [5].

**Fig. 8:**
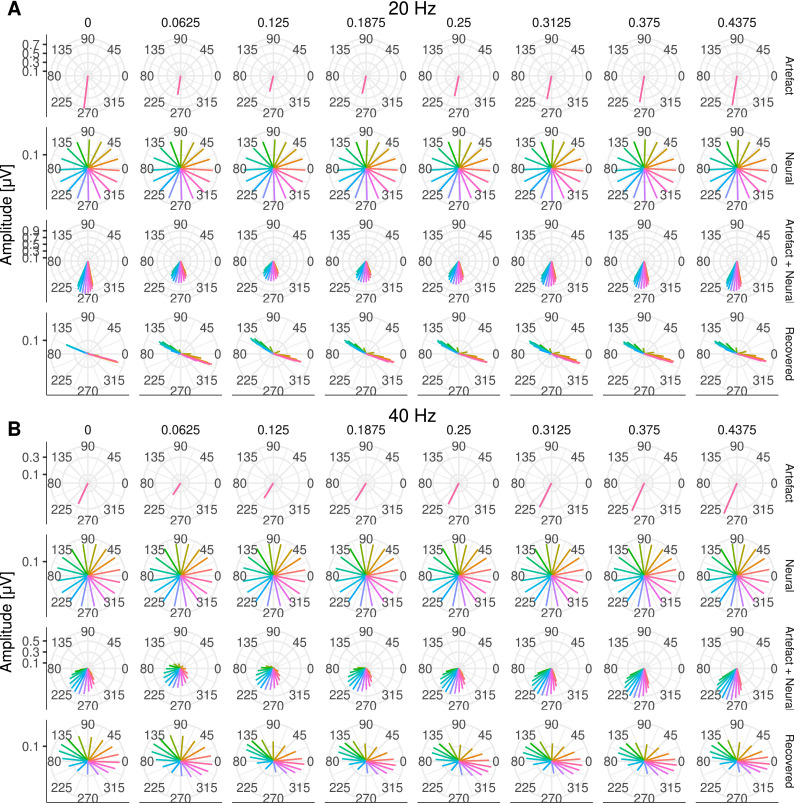
Amplitude and phase of ASSRs to each AM frequency at Cz. **A** for 20 and **B** for 40Hz. Each column corresponds to a different starting interpolation point (as a fraction of the pulse rate period). Each row shows the amplitude and phase of artefact only, target (neural), artefact plus neural, and recovered ASSR, respectively. The different phases of the neural source are colour coded.

**Fig. 9:**
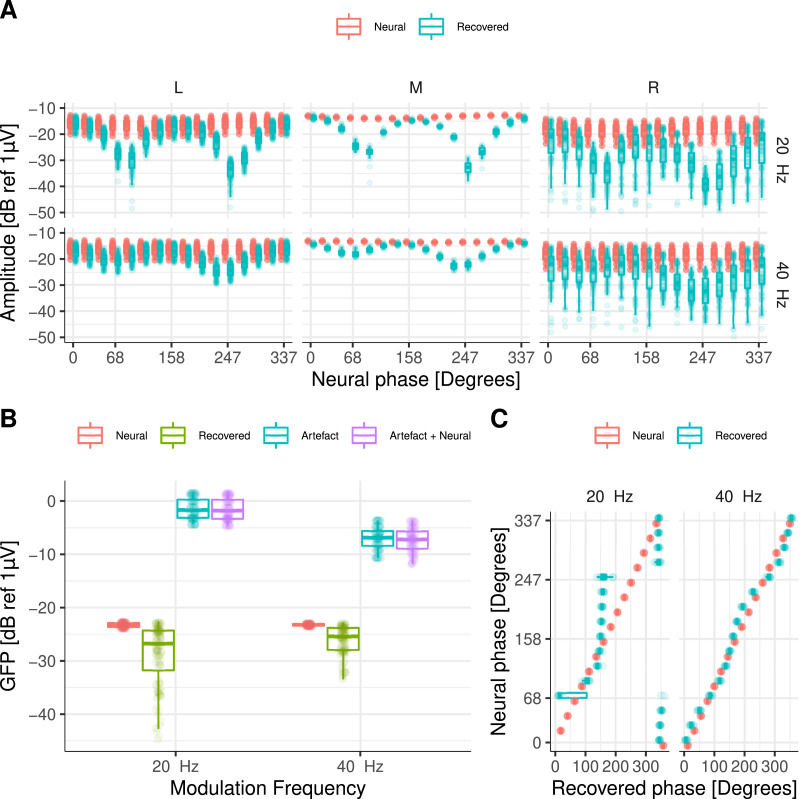
**A** Amplitudes across all frontal and central electrodes for target (neural), and recovered response. **B** GFP across all simulated conditions. Individual points correspond to different interpolation starting points and different neural phases. Target (neural), recovered response, artefact only, and artefact plus neural data are colour coded. **C** Target (neural) and recovered phase (in degrees) for simulated ASSRs to each AM frequency (indicated above each panel) at Cz. Individual points correspond to different interpolation starting points.

Next, we investigated the extent to which ASSRs to each AM embedded on the ACC response could be reliably recovered. This is illustrated in Fig. 8, where both the amplitude and the phase of the response to each AM frequency are shown at Cz. At 20 Hz (Fig. 8A), the electrical artefact (Artefact panel) was stronger than at 40 Hz (Fig. 8B). As in the previous section, it can be observed how the electrical artefact drags the neural response (Neural panel) towards its direction, distorting the phase and amplitude of the neural response (Arefact + Neural panel). By interpolating and removing the largest DSS components on the side of the CI, the amplitude of the recovered 20 Hz ASSR was reliably recovered for many of the simulated neural phases across recording electrodes (Fig. 9A). However, the phase of the recovered responses was distorted and had a direction orthogonal to the artefact (Fig. 8A and Fig. 9C). At 40Hz, both amplitude and phase (Fig. 9A & C) of the ASSR were reliably recovered across most conditions. Slight differences were only observed when the phase of the neural response was in the direction of the electrical artefact (Fig. 8B & Fig. 9A).

The estimated GFP to each simulated condition (Fig. 9B) indicated that at 20Hz, the average difference between the neural (−23.3dB ± 0.42dB) and the recovered GFP (−28.8dB ± 5.76dB) was 5.5dB, whilst at 40Hz, the average difference between the neural (−23.2dB ± 0.18dB) and the recovered GFP (−26.2dB ± 2.75dB) was 3.0dB.

As with the alternating AM frequency paradigm, we also evaluated the ability to extract ACC responses from artefact recordings to electrode changes at 902 pps. The results were identical, ACC responses were reliably recovered in all conditions.

These results demonstrate that interpolation and DSS artefact removal allow for successfully recovery of ACC and ASSRs. However, ASSR amplitude and phase may result distorted (smaller amplitudes and biased phases) as a result of the strong temporal correlation between the artefact and neural waveforms.

### Effectiveness of interpolation and spatial filtering to recover AC-FR

**Fig. 10:**
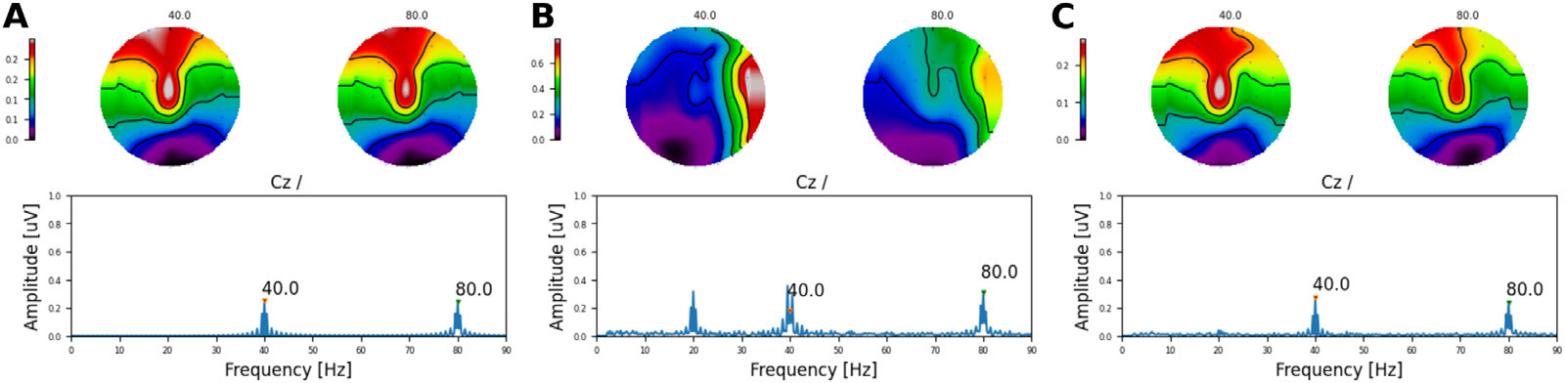
Topographic maps for AC-FR simulations at 902 pps. **A** Target neural response, **B** artefact plus neural response, and **C** recovered neural response. The amplitude is colour coded (in uV). Note that comodulations around the target frequencies are caused by the beating of each frequency (1Hz) within the analysis window used to compute the frequency-domain response (≈ 4 seconds).

Finally, we investigated the ability to recover AC-FRs from contaminated recordings (see Undurraga et al. [3] for details). Because we were unable to observe electrical artifacts related to the alternating rate for either electrode or AM frequency recordings in the cadaveric head, we recreated a highly contaminated AC-FR scenario using the data of the alternating AM frequency paradigm (20 and 40 Hz AM frequencies alternating at 1 Hz; 902 pps). Considering that potential residual artefacts (e.g. DC current caused be electrode changes, or accumulation of charge from one AM to another) should result in periodicities that are half the alternating rate, we simulated neural signals oscillating at twice the rate of the recorded AM frequency artefacts, i.e. we recreated a transposed version of the AC-FRs paradigm with neural sources oscillating at 40 and 80 Hz (Fig. 10A). As in the previous sections, we added the simulated steady-state neural data to the contaminated recordings (Fig. 10B). A total of four different interpolation points (between zero and one eight of the pulse rate period, before each pulse onset) and 16 different neural phases were simulated (64 different simulations).

**Fig. 11:**
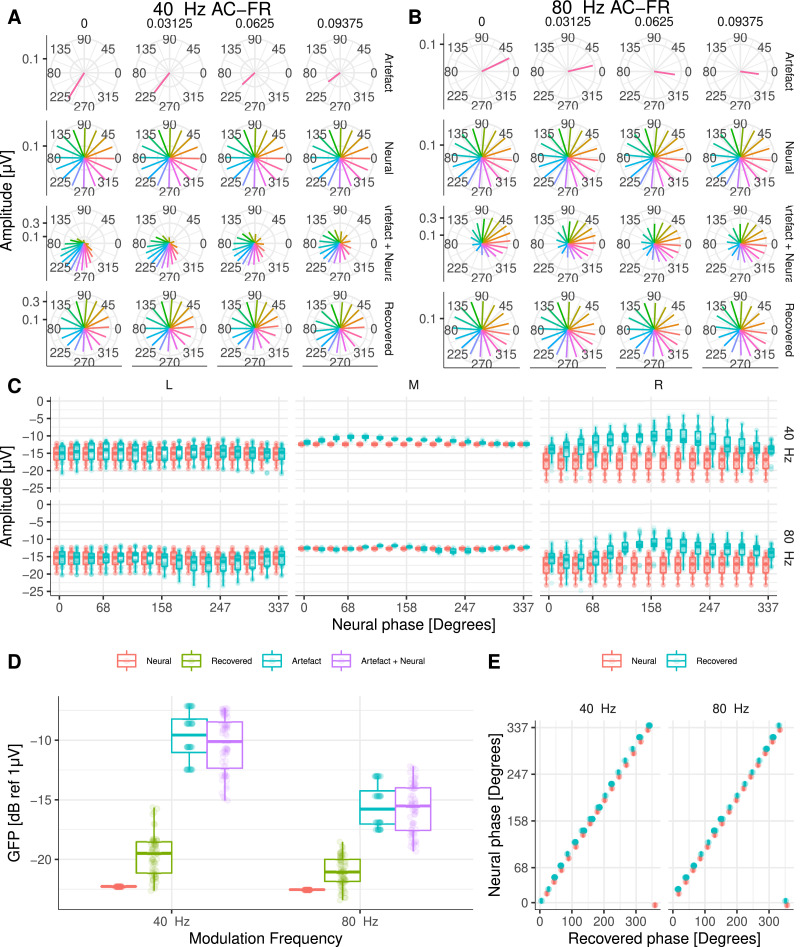
**A** and **B** Amplitude and phase of 40 Hz and 80Hz AC-FRs at Cz, respectively. Each row shows the amplitude and phase of: artefact only, target (neural), artefact plus neural, and recovered AC-FRs, respectively. The different phases of the neural source are colour coded. **C** AC-FR amplitudes for central and frontal recording electrodes on the left (L), middle (M), and right (R) side of the head. **D** GFP for all simulated AC-FRs. Target (neural), recovered, artefact only, and artefact plus neural data are colour coded. **E** Target (neural) and recovered phase (in degrees) for simulated AC-FRs at Cz.

The results demonstrated that we were able to recover AC-FRs in all conditions (Fig. 10C). Both phase and amplitudes were similar to that of the target neural source (Fig. 11A-E).

The estimated GFP to each simulated condition (Fig. 11D) indicated that at 40 Hz, the average difference between the neural (−22.3dB ± 0.005dB) and the recovered GFP (−19.6dB ± 1.68dB) was −2.7dB, whilst at 80Hz, the average difference between the neural (−22.5dB ± 0.005dB) and the recovered GFP (−21.0dB ± 1.22dB) was −1.5dB.

We also explored the ability to separate DC currents by means of a periodic squared pulse train as a “neural” source. The results were identical, and we could always recover amplitude and phase reliably. Our results demonstrate that AC-FR amplitude and phase can be recovered with minimal distortions. Deviations were observed only on the side of the CI (Fig. 11C), but these occurred when the phase of the artefact was similar to that of the neural response, an unlikely realistic expectation, since neural responses occur at a different timing than electrical artefacts, i.e. they have a different phase. Nevertheless, researchers should be aware that this may still be possible and should consider the extent to which the phase of the residual artefact overlaps with those reported in similar studies in normal-hearing (NH) listeners.

### Artefact removal in CI listeners

After assessing and validating the artefact removal method in the cadaveric specimen, we obtained recordings and removed electrical artefact from alive human CI users. The processing pipeline compromised the same steps described in this study, but included two additional steps: the removal of bad electrodes (usually electrodes with voltage offsets greater than 40 mV or strong DC components), and eye-blink artefacts suppression using a template matching method [11]. Participant details and specific stimulation parameters can be found in the companion publication [3].

#### Transient ACC responses to AM alternation

**Fig. 12:**
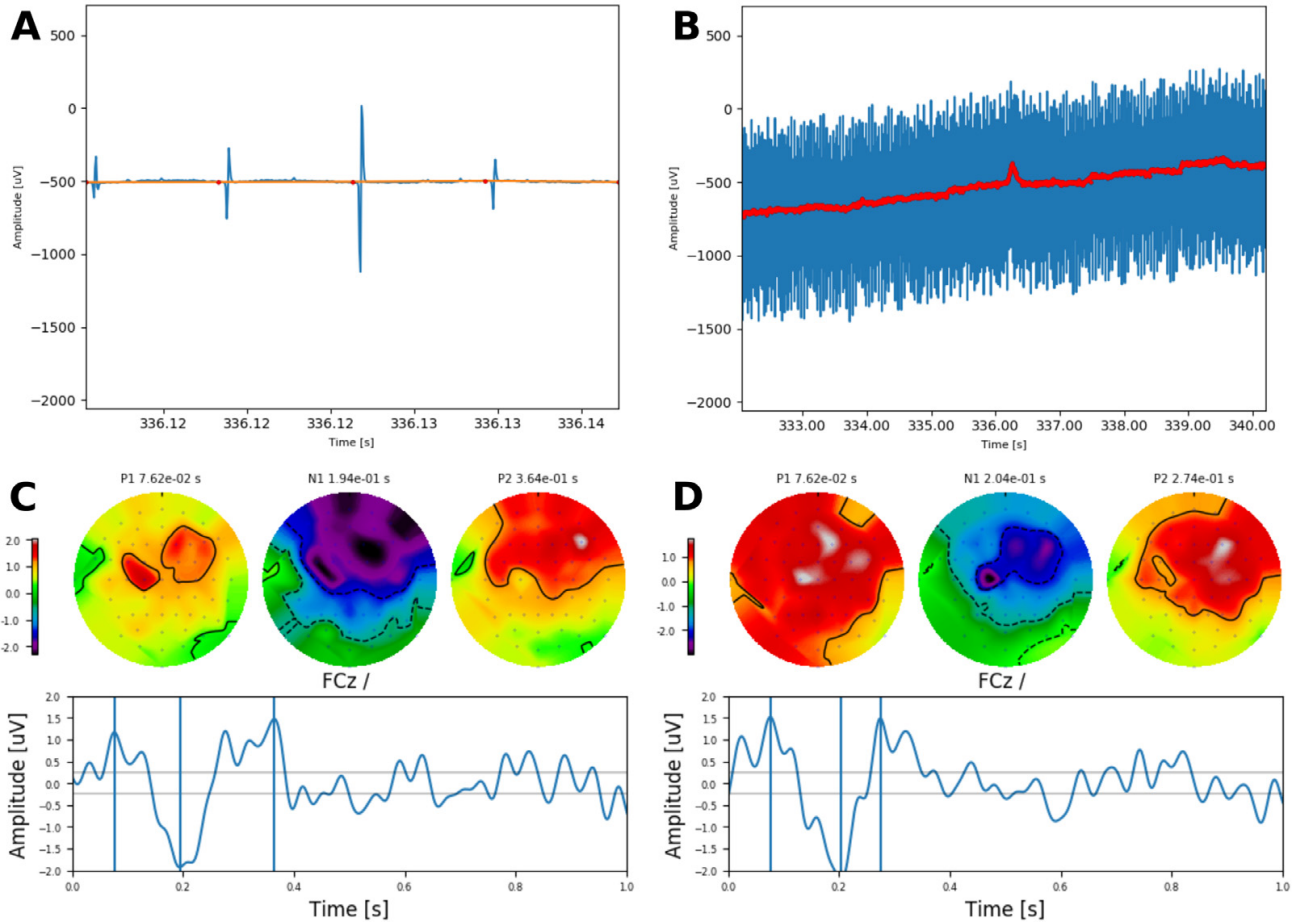
Artefact rejection via interpolation from subject S3. **A** Original recording in blue, interpolation points in red, and resulting waveform in orange. Two different time scales are shown in **A** and **B**. Example transient ACC responses to AM frequency changes imposed on a 126 pps QPC pulse train presented on electrode 16 to full (100%) modulated **C**, and halfway (50%) modulated **D**. The respective P1-N1-P2 peaks are shown by the vertical lines. The topographic map of each peak is shown on top where the amplitude is colour coded (in uV).

To illustrate the use of interpolation at low pulse rates, Fig. 12A & B shows the application of interpolation and the successfully removal of the electrical artefact whilst keeping the overall envelope of the EEG recording. Transient ACC responses (Fig. 12C & D) evoked by alternating the AM frequency imposed to a low pulse rate train (126 pps) between 20 and 35Hz at a rate of 0.5Hz were clearly observed, demonstrating a typical P1-N1-P2 morphology.

#### Artefact cancellation to electrode and AM frequency alternations at near-speech rates CI listeners

**Fig. 13:**
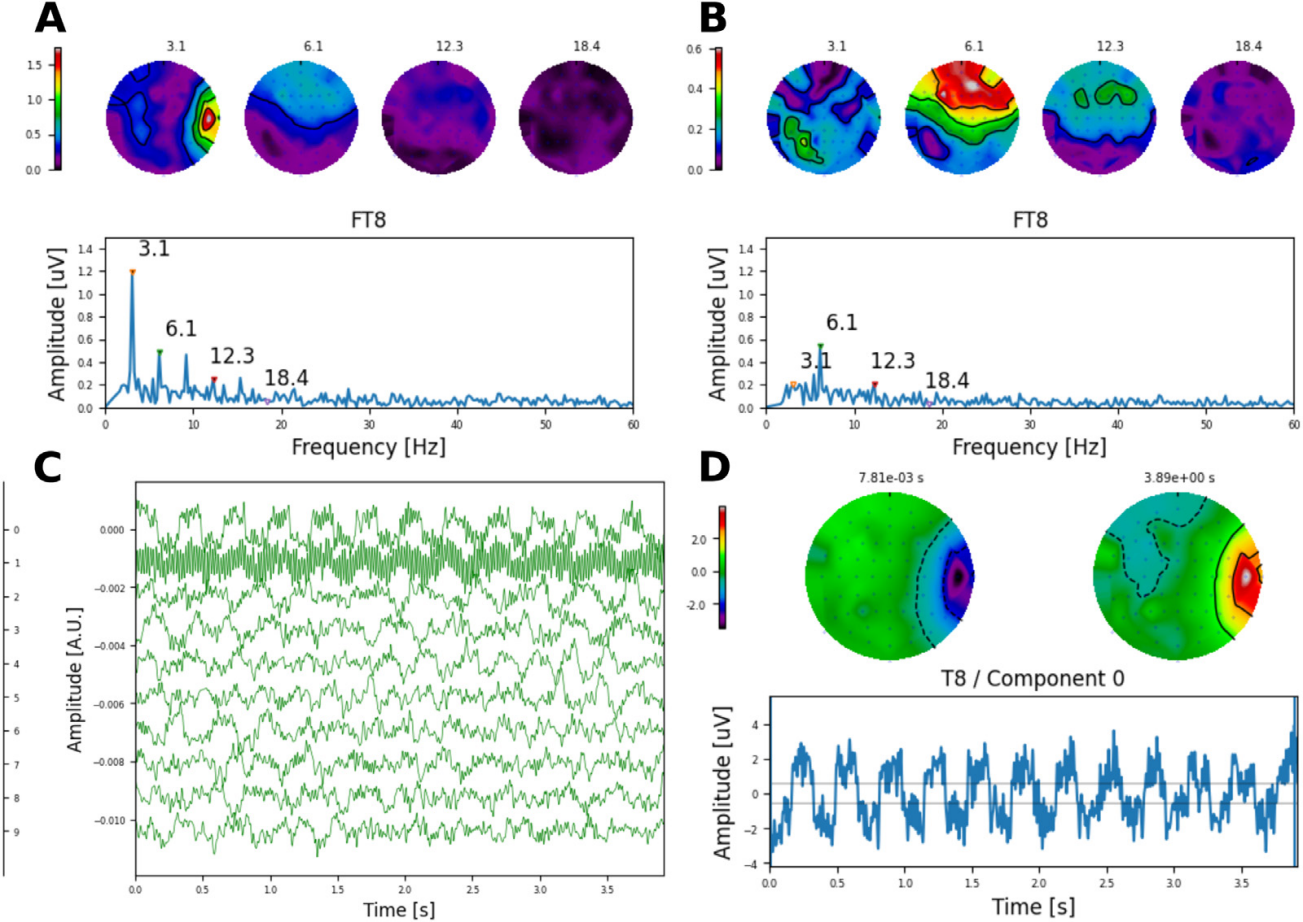
Removal of electrical artefacts caused by electrodes 16 and 17 alternating at a high rate (6.1 Hz) using 827 pps pulse trains in subject S2. **A** Shows the original waveform, and **B** the recovered AC-FR after removing the first two DSS components shown in **C**. Topographic maps show the amplitude of each frequency component indicated by the triangular markers (in uV). **D** Illustrates the projection of first DSS component on the sensor space. Topographic maps at the beginning and the end of the waveform show clearly that this component is caused by the CI (located on the right ear).

**Fig. 14:**
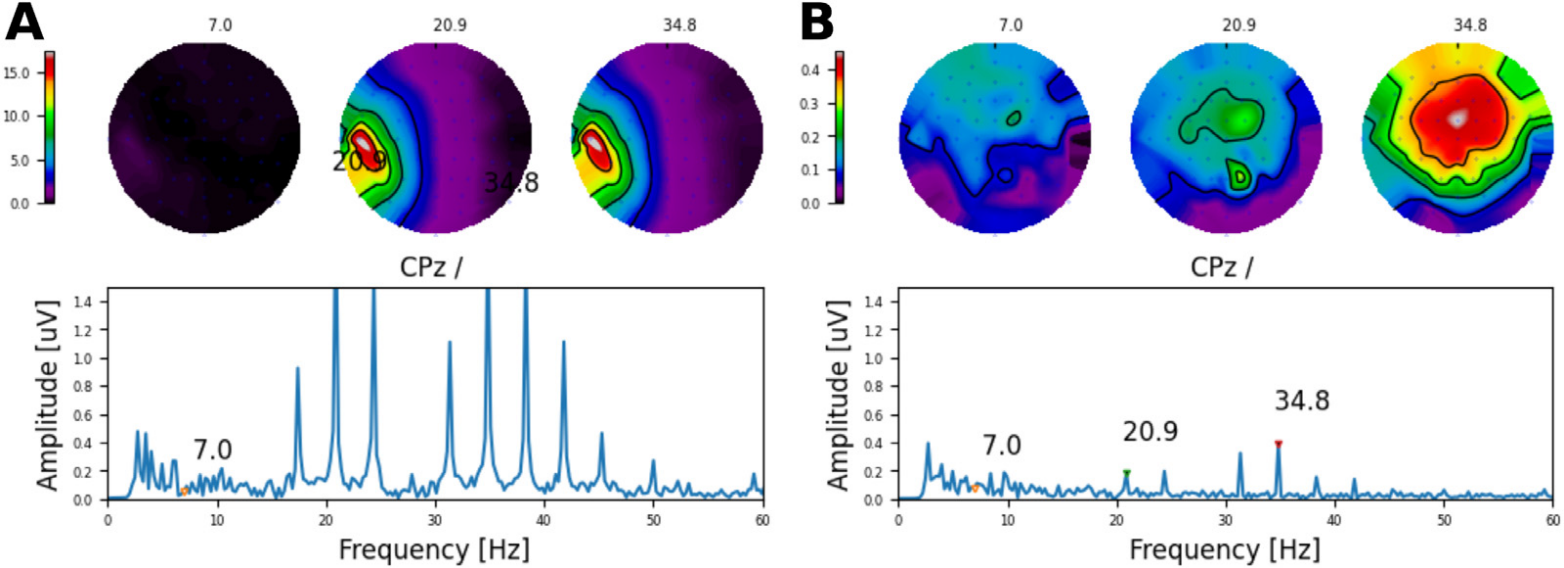
Removal of electrical artefacts caused by AM frequencies alternating at a high rate (6.9 Hz) on electrode 16 using 801 pps pulse trains in subject S3. **A** Shows the original waveform, and **B** the recovered response after removing the first DSS components where clear ASSRs to each AM frequency (20.9Hz and 34.8Hz) are observed. Topographic maps show the amplitude of each frequency component indicated by the triangular markers (in uV).

In this section, we provide real examples of artefact removal via interpolation and DSS at near-speech alternating rate using either the electrode (6.1 Hz; Fig. 13) or the AM frequency paradigm (6.9 Hz; Fig. 14) evoking AC-FRs.

For the electrode paradigm, obtained at a pulse rate of 827 pps, the spectrum of the contaminated response (Fig. 13A) shows multiple frequency components below and above the alternating rate, with a clear topographic bias towards the CI side (right ear) for the 3.05Hz frequency component. The artefact free response (Fig. 13B), was obtained by removing the first two components (Fig. 13C) and it is observed that the AC-FR to the alternating rate is clearly preserved, whilst the 3.05Hz component, caused by DC artefact from the alternation between electrodes (Fig. 13D), was successfully removed.

Similarly, we removed the electrical artefact recorded when the AM frequencies (≈ 20 and 35 Hz) alternated at 6.9 Hz using high pulse rate trains (801 pps). The CI related artefacts (Fig. 14A) were spatially biased towards the side of the CI (left ear), whilst the artefact free response (Fig. 14B), showed the expected topographic distribution. Note that neither for the electrode nor the AM paradigm, the response amplitude at the alternating rate was affected by our method, in line with the results of the simulations. Importantly, the topographic maps of the recovered responses showed a distribution which is consistent with that of a neural source and similar to that of the ACC using low alternating rates (Fig. 12).

A complete set of measurements in CI listeners applying the methods described in this article, and using several stimulation paradigms (ACC, ASSRs, and AC-FRs) can be found in the associated research article [3].

### Summary and conclusions

The results, where realistic artefacts were obtained from an implanted human head and alive CI listeners, provide strong evidence that combining interpolation and DSS can significantly reduce CI artefacts. ACC and AC-FRs could always be recovered at high (via interpolation and DSS) or low (via interpolation) pulse rates, whilst ASSRs could be reliably recovered at low pulse rates (via interpolation). At high pulse rates, the recovered ASSR (via interpolation and DSS) had either similar or smaller amplitudes than the neural source. The results also demonstrated that, regardless amplitude-phase distortions, topographic maps are well preserved. Thus, topographic maps provide valuable information to assess the nature of the recovered response. We also found that ASSR amplitude and phase should be carefully assessed. For example, if the phase of the recovered response were orthogonal to that of the contaminated response, this would suggest that the phase and amplitude have been distorted. Conversely, if the phase of the recovered response is not orthogonal to that of the contaminated response, the phase may have been properly recovered, but the amplitude could be smaller than that of the neural source. However, it is very unlikely to expect that the neural response will have the same phase than the electrical artefact due to implicit neural delays associated to a neural response. In either case, our results indicate that the presence of the ASSR (after artefact removal) is indicative of a true neural response, whilst the lack of it does not proof its absence.

## Declaration of Competing Interest

The authors confirm that there are no conflicts of interest.

## Appendix A Supplementary materials

Supplementary Data S1 Supplementary Raw Research Data. This is open data under the CC BY license http://creativecommons.org/licenses/by/4.0/ Supplementary Data S1

Supplementary Data S2 Supplementary Raw Research Data. This is open data under the CC BY license http://creativecommons.org/licenses/by/4.0/ Supplementary Data S2
